# Willingness to Pay for a Contributory Social Health Insurance Scheme: A Survey of Rural Residents in Akwa Ibom State, Nigeria

**DOI:** 10.3389/fpubh.2021.654362

**Published:** 2021-06-17

**Authors:** Christie Divine Akwaowo, Idongesit Umoh, Olugbemi Motilewa, Bassey Akpan, Edidiong Umoh, Edidiong Frank, Emmanuel Nna, Uchenna Okeke, Obinna E. Onwujekwe

**Affiliations:** ^1^Department of Community Medicine, University of Uyo, Uyo, Nigeria; ^2^Department of Internal Medicine, University of Uyo, Uyo, Nigeria; ^3^Department of Geography and Natural Resources, University of Uyo, Uyo, Nigeria; ^4^Obstetrics and Gynaecology, Immanuel Hospital, Eket, Nigeria; ^5^The Molecular Pathology Institute, Enugu, Nigeria; ^6^Department of Radiology, Nigerian Navy Reference Hospital, Calabar, Nigeria; ^7^Department of Pharmacology and Therapeutics, Institue of Health Policy and Research, University of Nigeria, Enugu, Nigeria

**Keywords:** contingent valuation, health insurance, rural residents, universal health coverage, willingness to pay, Nigeria

## Abstract

**Background:** Health insurance is seen as a pathway to achieving Universal health coverage in low- and middle-income countries. The Nigeria Government has mandated states to set up social health insurance as a mechanism to offer financial protection to her citizens. However, the design of these schemes has been left to individual states. In preparation for the set-up of a contributory social health insurance scheme in Akwa Ibom State, Nigeria. This study assesses the willingness-to-pay for a social health insurance among rural residents in the state.

**Methods:** The study was conducted in three local government areas in Akwa Ibom State, South south Nigeria. It was a cross-sectional study with multi-stage data collection using a demand questionnaire. Interviews were conducted with 286 household heads who were bread winners. Contingent valuation using iterative bidding with double bounded dichotomous technique was used to elicit the WTP for health insurance. Multiple regression using least square method was used to create a model for predicting WTP.

**Findings:** About 82% of the household heads were willing to pay insurance premiums for their households. The median WTP for insurance premium was 11,142 Naira ($29), 95% CI: 9,599–12,684 Naira ($25–$33) per annum. The respondents were predominantly middle-aged (46.8%), Ibibio men (71.7%) with an average household size of five persons and bread winners who had secondary education (43.0%) and were mainly pentecostals (51.5%). The mean age of respondents was 46.4 ± 14.5 yrs. The two significant predictors of WTP for insurance premium amongst these rural residents were income of breadwinner (accounts for 79%) and size of household (2%). The regression coefficients for predicting WTP for insurance premium are intercept of 2,419, a slope of 0.1763 for Bread winner income and a slope of 741.5 household size, all values in Naira and kobo.

**Conclusion:** Majority of rural residents in Akwa Ibom State were willing to pay for social health insurance. The amount they were willing to pay was significantly determined by the income of the breadwinner of the household and the size of the family. These findings are relevant to designing a contributory social health insurance scheme that is affordable and sustainable in order to ensure universal health coverage for the citizens.

## Introduction

Universal Health Coverage is a global target that has been adopted by all nations, regions and states. The World Health Organization defines Universal Health Coverage (UHC) as a means of organizing, delivering and financing health services in such a way that ‘all people obtain the health services they need without suffering financial hardship when paying for them (1). It combines two attributes: first, everybody is covered by a package of good quality essential health services; and second, it provides financial protection from healthcare costs, especially at the point of service delivery (1, 2). Embedded in the definition of UHC is the idea of protecting people from financial hardships and social inequality.

The Nigerian healthcare system is currently undergoing major reforms in health care financing aimed at achieving UHC. The Federal Government of Nigeria has directed all states to set up and run mandatory State health insurance schemes (3, 4). To this end, many states of the federation that have launched the statewide scheme health insurance schemes (5). With the passage of the health insurance bill, the National Health Insurance Scheme (NHIS) has the mandate to protect all Nigerians from paying for healthcare out of pocket. However, to achieve UHC in Nigeria, an effective NHIS implementing the dictates of the bill is imperative.

Social health Insurance (SHI) is one of the possible organizational mechanisms for raising and pooling funds to finance health services, along with tax-financing, private health insurance, community insurance, and others (6). It improves access to health services by removing catastrophic health expenditures by pooling funds to allow cross-subsidization between the rich and poor and between the healthy and the sick (7, 8). Such schemes have evolved over the years to represent a variety of funding mechanisms, voluntary or involuntary, with the underlying objective that all people are, or will be over time, offered the right to enrolment in at least one type of mechanism allowing financial risks to be shared (7). This might involve a mix of various forms of insurance funding for some types of health services with others being funded directly from government revenues (9).

Under the Nigerian national health insurance scheme (NHIS), the SHI is designed to cover the formal sector and the informal sector. The formal sector scheme is currently providing cover for only 5% of the population (9). Setting up the informal sector schemes have been challenging because of the diversity of sector which includes the urban self-employed; rural community; children under-five; permanently disabled persons; prison inmates; tertiary institutions and voluntary participants; and armed forces, police and other uniformed services. The main challenge is how to generate adequate revenue to finance health services from a diversified group of people, without over tasking the formal sector workers.

Raising revenue for these schemes become even more challenging because of dwindling government revenue for the health sector and the large huge informal sector, infective tax collection system, inefficient formula to calculate the amount to collect (9). It is therefore particularly important to gather reliable information on the amount of money people are willing to pay for a social health insurance scheme.

In health utility and welfare economics, Willingness to Pay (WTP) is the amount in monetary value that a person would be ready to spend to utilize a healthcare service (10). The concept of willingness to Pay WTP for health insurance arises in settings where access to healthcare is mostly contingent on Out-Of-Pocket Spending (OOPS) at the point of service delivery. Even in insurance-based setting, health insurance schemes must know up front how much they could charge as premium, to ensure their financial sustainability (11).

Several methods of estimating WTP exist. A useful way of conceptualizing the measure of WTP is the hierarchical classification framework. This organizes existing methods of WTP estimation based on data collection methods as proposed by Breidert, Hahsler and Reutterer (12). Using this concept, there are broadly two approaches to estimating WTP: “Revealed Preferences” (RP) and “Stated Preferences” (SP) also referred to as Contingent Valuation (CV).

Contingent Valuation has been widely used to assess public preferences through eliciting WTP values (13, 14). The contingent valuation method (CVM) is grounded in welfare economics, with the measurement of consumer surplus providing the major theoretical underpinning (14). In order to ascertain the Hicksian surplus or willingness to pay (WTP) empirically, the CVM is often employed. It is a survey-based hypothetical method of determining economic values on public goods by obtaining information on individual preferences. It determines what they would be willing to pay for public goods and services when prices are not available.

The CV method assumes that prospective clients are given a detailed description of the product for which they are asked how much they would be willing to pay for certain goods using a bidding game. This bidding game is useful for eliciting WTP in the Nigerian setting as most goods are purchased using the haggling method in the open market (14). Many studies in low and middle income countries have found that the CVM is a valid technique for valuing the maximum amounts of money that people are willing to pay for healthcare goods and services (14–19). Most of these studies have attempted to elicit factors associated with WTP (20, 21).

Some studies on WTP for health insurance have been carried out in various parts of Nigeria (14, 22–26). Majority of these studies report a high rate of willingness to willingness to participate and pay for health insurance, ranging from (25, 27–29). However, there are also studies reporting low willingness to pay among respondents (30, 31). Similarly a study carried out among health professionals in Ethiopia reported a 28.7% willingness to participate rate (31).

The factors associated with WTP are many and vary with context. However, factors that were found to consistently exert a positively influence on WTP include male gender (18, 28, 30), educational status (18, 25, 27, 28, 31), income (16, 25, 29, 30), household size (32), socioeconomic status (30), family size and ability to pay (18). Previously paying for any form of health insurance and previous experience of ill health were also positive factors for WTP (30, 33).

However, previously paying out of pocket was negatively associated with WTP (30). Other factors that negatively /inversely related to WTP include chronic illness (28, 34).

Although several studies on the willingness to pay (WTP) for health insurance schemes have been carried out amongst different sectors of the society, majority have been done among the urban dwellers and the informal sector (5). There is a paucity of data on the WTP for health insurance by rural dwellers. This study documents the first WTP estimation carried out in Akwa Ibom state. The purpose of our study *was to* determine the willingness to pay for health insurance amongst rural dwellers in Akwa Ibom State, Nigeria.

## Materials and Methods

### Objective

The main objective of this study was to estimate the Willingness to Pay for health insurance among rural households in Akwa Ibom State using a double-bounded discrete choice analysis.

### Study Design

This is a cross-sectional study with multi-stage, non-probability sampling method. An instrument, using several questions, scenarios and iterative bidding was administered to respondents using the Open Data Kit (ODK) software tool (35).

### Study Setting

Akwa Ibom State is located in the coastal land of South East Nigeria. It has a population of about 5.48 million people and a land area of 7,081 squared km (36). It has an urban-rural dweller ratio of 3:2. Majority of both urban and rural dwellers have no health insurance coverage. Currently, the State has not started the mandatory health insurance scheme.

Majority of both urban and rural dwellers have no health insurance coverage. Currently, the State has not started the mandatory health insurance scheme. There is paucity of data on the WTP for health insurance by rural dwellers.

Akwa Ibom state is located in the South South geopolitical zone of Nigeria, lying between latitudes 4^o^321'N and 5^o^331 North, and longitudes 7^o^251 and 8^o^251 East. It is bounded by Cross River State to the East, Abia to the North, Abia and Rivers to the West, and the Atlantic Ocean to the South. The capital of the state is Uyo, with about 1,200,000 inhabitants (36, 37). This is an oil-rich state comprising 31 local government areas (LGAs), stratified into urban, semi-urban and rural.

The state is politically subdivided into three senatorial districts- Uyo, Eket and Ikot Ekpene. Each of the zones is made of 10–12 LGAs. The state is primarily agrarian in nature, and the rural area practice mostly subsistence farming. However, those in the coastal areas are into fishing. The state is predominantly populated by the Ibibios, Annangs, and the Oron. The main populace are the Ibibios, other ethnic groups in the state are the Ibenos, and the Obolos, who are domiciled in the coastal parts of the state. However, a lot of foreign citizens from all over Nigeria reside in the state capital city, Uyo. Majority of both urban and rural dwellers have no health insurance coverage because the State has not started the mandatory health insurance scheme.

### Study Sites, Sample Size and Sampling Method

The survey was conducted in three selected local government areas (Ibiono, Essien Udim and Esit Eket) of Akwa Ibom state. For the household survey, sample was determined using Raosoft Calculator for estimating single proportions in a population survey (38). Using a standard normal deviate at 95% confidence level, 5% margin of error, an estimate of possible true proportion of 20%, the sample size was 243 at 80% power. A 10% non-response rate was added to obtain 267 respondents, but the eventual sample size was 286.

In each of the three LGAs, enumeration areas were randomized to select communities. Selected communities were randomized for households. Heads of households were sampled by non-probability method.

### The Instrument

The instrument used is titled the Demand Questionnaire. Table 1 shows the components of the demand questionnaire. It comprised the following sections: Section A: Consent form, Identification sheet; Section B: Household Health Seeking Behavior; Section C: Satisfaction with Existing Public Services; Section D: Income and Household Expenditure on Primary Care; Section E: Demand Assessment for Health Cooperatives and Section F: Interest in Prepaid Health Insurance and G: Contingent valuation to elicit WTP for health insurance. The instrument was developed specifically for the study.

**Table 1:** Components of the Demand Questionnaire.

**Section**	**Title**
Section A	Consent form & identification sheet
Section B	Household health seeking behavior
Section C	Satisfaction with existing public services
Section D	Income and household expenditure on primary care
Section E	Demand assessment for health cooperatives
Section F	Interest in prepaid health insurance
Section G	Contingent valuation to elicit WTP for health insurance.

Section A contained a Consent form where each respondent was informed-consented. It also had an identification section that measured respondent's demographics such as age, sex, ethnicity, religion, position in the household, number of people in the household and educational status of the head of household.

Section B contained the Household Health Seeking Behavior which included course of action taken when a household member was ill, types of medications being currently taken, who was responsible for paying for treatment, where the ill person was taken to for treatment and when last a household member fell ill.

Section C contained satisfaction with existing public services which included satisfaction with waiting time in the government health facilities, friendliness of staff in the facilities, competence of the health workers in explaining health situations, proximity of the health facility, ambience of the facility, availability of diagnostic services and drugs and satisfaction with range of services offered.

Section D is income and household expenditure, which included monthly income of the household head and spouse, amount spent for the entire household for treatment per month, how payment for treatment affected feeding, frequency of borrowing money to treat ill member of the household, how long it took to repay the loan and perceived health status of the respondent.

Section E was Interest Assessment for Health Cooperatives, which included monthly contributions to any contribution group, how long the person had participated in the contribution group and level of trust in the contribution group; assessed if the person had belonged to any co-operative in the past, and whether they trusted management of co-operative and if they are still active in co-operatives. It also assessed types of co-operatives: (a) normal co-operative with all incentives, (b) health co-operative, (c) health plus-co-operative.

Interest in taking health insurance (Section F of the instrument) was determined by creating hypothetical scenarios describing proposed service options for a publicly provided social health insurance scheme and people's willingness to join the schemes was assessed.

For the WTP (Section G), a contingent valuation approach was used to determine the willingness to pay. For this survey, a double-bounded dichotomous choice elicitation method was used.

### Scenario

The respondents were presented with a hypothetical scenario describing a potential State Health Insurance product. We proposed a hypothetical scenario, explaining the government's intention to set up SHIS in order to improve access for health care. We presented the different attributes of the health insurance scheme.

“*The Federal Government of Nigeria has mandated all states in the country to set up state health insurance schemes in order to provide better access to quality health services for their citizens. The Akwa Ibom State Government has made efforts to actualize this by signing into an Act of law the Akwa Ibom State Health Insurance Bill. We are gathering information to help the government to commence health insurance scheme for all peoples in the state. Please help us by answering the following questions:*

“*Assuming you are invited to be a part of a health insurance scheme that you:*

- *Can access quality health care for yourself and family at any time*
- *Must make regular contributions to (monthly, weekly)*
- *Don't have to pay full cost for service at point of use*
- *Can make regular contributions for* ***ONLY****health services*
- *Can assist to pay for others who cannot afford to pay for health care in your community?*
- *You can have most illnesses covered but* ***not EVERYTHING.”***

To reduce the limitation of starting point bias in the bidding game, four different starting bids were used. The values were obtained after a pre-test and consultation with the personnel in the Community health insurance scheme (CBHIS) in the state. The double-bounded dichotomous choice elicitation method was used because it is known to increase statistical efficiency gains (13). After identifying the initial bids, the respondents were asked whether they were willing to pay or not. For instance, if he or she said “yes” to the first bid, a second higher bid was given and if he or she says “No” to the first bid, the second lower bid was asked. If he or she said “no” to both the first and the second bids, then the person was asked the maximum that he or she was willing to pay.

In considering the contingent valuation method limitation of starting point bias, this study reduces this (bias) by using the four different starting bids. The initial bids are the following amounts: 10,000, 15,000, 20,000, and 25,000 Naira per year, which were 26, 39, 53 and 66 USD, respectively (exchange rate of N380 per dollar) and this bid were randomly assigned to the respondents as adopted by Onwujekwe et al. (30). Following the first answer, the second bid amount will be reduced by half if response in the first bid is “No” and doubled if answer to the first bid is “Yes”.

### Ethical Approval

Ethical approval was given by the Institutional Health Research Ethics Board of the University of Uyo Teaching Hospital, Akwa Ibom State. The ethics approval number is **UUTH/AD/5/96/VOLXXI/480**.

### Data Collection

Administrators of the instruments gathered data using the ODK Collect app on their phones. This is an open-source Android app that replaces paper forms used in survey-based data gathering. It supports a wide range of question-and-answer types and is designed to work well without network connectivity (35).

### Data Analysis

Descriptive statistics was used to summarize demographics and various measures of interests, house income and satisfaction. We performed multiple linear regression using ordinary least square method (OLS) to establish a regression model for predicting WTP based on quantitative explanatory variables such as household income of head of household (breadwinner) and spouse, age of the head of household, household health expenditure and number of people in a household. Significance level was set at 5%. All analyses will be performed using GraphPad Prism version 9.0.

## Results

A total of 286 household heads were studied. Socio-demographics of respondents is shown in Table 2. The respondents were predominantly middle-aged (46.8%), Ibibio men (71.7%) with an average household size of five persons, who had secondary education (43.0%) and were mainly Pentecostals (51.5%). The mean age of respondents was 46.4 years with a standard deviation of 14.5 yrs. Only 86 (30.1%) had heard of health insurance, and only 7 (2.5%) belong to a health insurance scheme.

**Table 2:** Demographics of respondents (*N* = 286).

**Variable**	**Number**	**Percentage**
**Gender of head of household**		
Male	205	71.7
Female	81	28.3
**Position of respondent in the household**		
Head of Household	201	70.3
Spouse	85	29.7
**Education status**		
No formal education	10	3.5
Primary education	78	27.3
Junior Secondary education	22	7.7
Senior Secondary education	123	43
Vocational certificate	3	1.1
Diploma/NCE	14	4.9
HND/Graduate	29	10.1
Postgraduate	7	2.5
**Ethnic group**		
Ibibio	213	74.5
Annang	67	23.4
Oron	3	1.1
Efik	2	0.7
Others	1	0.4
**Religion of head of household**		
Catholic	36	12.6
Anglican	65	22.7
Pentecostal	146	51.1
Traditional	0	0
Others	39	13.6
Age of household head (mean ± SD) years	46 ± 15	NA
No household residents (mean ± SD)	5 ± 3	NA

Respondents' satisfaction with public health facilities is shown in Tables 3, 4. Of the 286 respondents, 79.6% were satisfied with the waiting time in the public facilities while 79% agreed that the staff in public hospitals were friendly.

**Table 3:** Respondents satisfaction with public health facilities.

**Waiting time**	**Count**	**Percent**
Strongly agree	55	23.6%
Agree	131	56.2%
Neither agree nor disagree	20	8.6%
Disagree	21	9.0%
Strongly disagree	6	2.6%
**Friendliness of staff**		
Strongly agree	52	22.3%
Agree	132	56.7%
Neither agree or disagree	22	9.4%
Disagree	26	11.2%
Strongly disagree	1	0.4%
**Staff offered good explanation**		
Strongly agree	51	21.9%
Agree	139	59.7%
Neither agree nor disagree	23	9.9%
Disagree	19	8.2%
Strongly disagree	1	0.4%
**Knowledgeable staff**		
Strongly agree	50	21.5%
Agree	146	62.7%
Neither agree nor disagree	16	6.9%
Disagree	19	8.2%
Strongly disagree	2	0.9%
**Staff attention time**		
Strongly agree	55	23.6%
Agree	144	61.8%
Neither agree nor disagree	16	6.9%
Disagree	16	6.9%
Strongly disagree	2	0.9%
**Diagnostic test available**		
Strongly agree	42	18.0%
Agree	138	59.2%
Neither agree nor disagree	20	8.6%
Disagree	27	11.6%
Strongly disagree	6	2.6%

**Table 4:** Satisfaction of respondents with public health services.

**Drugs available**		
Strongly agree	40	17.2%
Agree	133	57.1%
Neither agree nor disagree	18	7.7%
Disagree	39	16.7%
Strongly disagree	3	1.3%
**Happy_with_service_range**		
Strongly agree	40	17.2%
Agree	136	58.4%
Neither agree nor disagree	31	13.3%
Disagree	25	10.7%
Strongly disagree	1	0.4%
**Like the facility**		
Strongly agree	46	19.7%
Agree	126	54.1%
Neither agree nor disagree	35	15.0%
Disagree	24	10.3%
Strongly disagree	2	0.9%
**Facility is near**		
Strongly agree	52	22.3%
Agree	123	52.8%
Neither agree nor disagree	9	3.9%
Disagree	44	18.9%
Strongly disagree	5	2.2%
**Access to referral center**		
Strongly agree	11	4.7%
Agree	39	16.7%
Neither agree nor disagree	100	42.9%
Disagree	59	25.3%
Strongly disagree	24	10.3%

Table 5 shows the distribution of the median WTP, median income of the breadwinner in the household, average amount spent on health among respondents. The median amount spent on health was found to be increasing with rising median breadwinner income.

**Table 5:** Median values of WTP across covariates in multiple regression.

**Median WTP (Naira)**	**Median Income of Bread winner (Naira)**	**Sample size per median income band**	**Median amount Paid for Health per family**	**Median age of head of family**	**Median Size of household**	**Median income of spouse**
5,000	10,000	109	500	45	5	10,000
10,000	30,500	119	3,000	45	5	10,000
10,000	50,500	43	3,000	45	5	30,500
10,000	70,500	7	3,000	43	2	30,500
17,500	90,500	6	5,250	55	6.5	30,500
42,500	130,000	2	5,250	50	7.5	20,250

The willingness to pay (WTP) for health insurance premium for the rural residents whose breadwinner income ranges from 10,000–130,000 naira ($26–342), was 11,142 naira ($29) with a 95% CI (9,599–12,684 naira or $25–$33) per annum. Majority of the respondents (82%) were willing to pay for health insurance premium.

**Figure 1:**
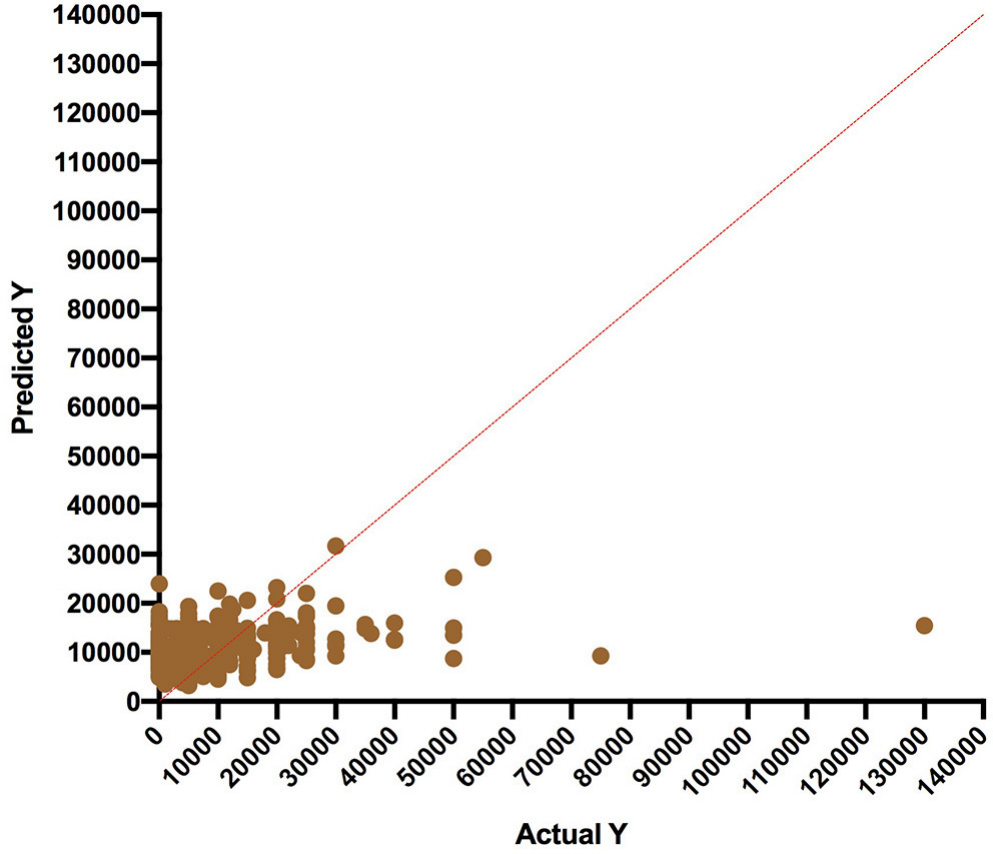
Actual vs. predicted WTP using a multiple linear regresion.

Figure 1 shows the regression model comparing the predicted WTP and actual WTP values. The clustering (agreement) of data points (predicted vs. actual) was strongest in breadwinner median income range of 10,000–30,500 Naira, which contained 80% of the respondents. The regression model showed that 79% of WTP was determined by the breadwinner income while the size of the household contributed to 2% in predicting WTP. The Regression equation is shown in section 4.9. Of the five quantitative variables assessed, only the breadwinner income and size of household were statistically significant in predicting WTP (*p* values were 0.0001 and 0.009, respectively). Spouse income, age of the head of the family and amount paid for healthcare per household were not significant predictors. Multicollinearity was checked and was not present.

## Discussion

In this paper we present the findings of a contingent valuation survey aimed at determining the willingness to pay for social health insurance amongst rural dwellers in Akwa Ibom State, Nigeria. Only 86 (30.1%) had heard of health insurance, and only 7 (2.5%) belong to health insurance scheme. Majority of respondents (82%) were willing to pay for a contributory health insurance scheme. The amount that households whose breadwinner income ranged from N10,000–130,000 naira ($26-−342), was 11,142 naira ($29) per annum. Predictors of WTP were breadwinner income and size of household.

Our study shows that only 86 (30.1%) respondents were aware of health insurance, and only 7 (2.5%) belong to health insurance scheme. Studies carried out in other low middle income countries show that a lot of people are not aware of health insurance (17). Similarly, studies in states in Nigeria have reported low levels of awareness, only (28.9%) in Ilorin and 28.7% in Abakaliki of the respondents had heard of health insurance before (25, 27, 39).

Majority of the respondents in the rural areas were willing to pay for a contributory health insurance scheme. Similar findings had been reported in other LMICs like Sierra Leone and Ethiopia (17, 18). Studies carried out in states in Nigeria have also reported high levels of WTP with 87% in Osun, 82% in Kaduna, 89.7% in Port Harcourt (24, 25, 27, 29). However, an older study on WTP for insecticidal treated nets reported a lower percentage of 40% (30). A study of WTP among health professionals in Ethiopia reported that only 28.7% of them were willing to pay, citing factors such as “thinking the government should cover the cost,” preferring out-pocket payment and that the provided SHI scheme does not cover all the health care costs (31). Our study findings showed that majority of rural residents in Akwa Ibom State were willing to pay for a social health insurance.

The amount the respondents were willing to pay was similar to findings from other studies (27, 39). However, some studies had reported lower amounts (24, 25). In a study of WTP among self-employed people in Port Harcourt, the average amount a person was willing to pay for a social health insurance was ₦539.22 ± 413.8 ($1.5 USD note that exchange rate and inflation rate have changed since then). In a study in Osun State, where nearly 83% household heads were more willing to pay for a community based health care financing scheme, they reported a WTP of ₦1798.90k per year amongst urban dwellers while rural dwellers were willing to pay ₦721.70k (23).

The amount that households whose breadwinner income ranged from N10,000–130,000 naira ($26–342), was 11,142 naira ($29) per annum. The amount found in our study is less than the stipulated 15,000 naira for the informal sector health insurance scheme as recommended by NHIS (5). This emphasizes the need for cost sharing and subsidization of the rural people by the government.

Other factors affecting WTP include place of residence and income level. Onwujekwe et al. found that less people living in the rural areas were willing to pay for health insurance (30). This was contrary the findings of Usman that rural dwellers were more willing to pay (28). Factors associated with increased willingness to pay include income level. This reflects the ability to pay. In the study by Gidey et in Ethiopia, respondents' WTP was signifcantly positively associated with their level of income but their WTP decreased with increasing age and educational status (16).

Predictors of WTP have been vary depending on the sector of the society being studied and the place. Studies in Burkina Faso and Ethiopia reported predictors of WTP as household and individual ability-to-pay, household and individual characteristics, such as age, sex and education (17, 18). In the Port Harcourt study, the predictors of WTP were marital status, level of education and mode of payment of healthcare (25). In our study, we found that WTP was predicted mostly by breadwinner income and size of household.

The differences observed between the studies could be due to the level of satisfaction with the public health facilities in studied area and occupation. It is plausible that the WTP observed in these respondents are influenced by satisfaction with services from health facilities and friendliness of staff amongst other variables. Our study found out that income of spouse, age of household head and amount previously paid for family healthcare were not significant predictors of WTP.

The policy implication of these findings is that a state-run social contributory health insurance scheme SHIS will be accepted by majority of rural dwellers in Akwa Ibom State. This study also shows the amount the residents in the rural areas would be willing to pay was significantly linked to breadwinner income and size of household. By implication, an improvement in breadwinner income will lead to increase in WTP in the rural areas. This will reduce the cost share and burden of financing the health sector by the government.

### Limitations of the Study

This study was solely on rural residents. It did not study urban residents whose socio-demographics and income level may differ markedly. The study only focused on a publicly provided model of health insurance not private schemes.

## Conclusion

This study presents results of an exploratory investigation using the double bounded dichotomous method of a contingent valuation to determine the willingness to pay for social health insurance amongst rural dwellers in Akwa Ibom State, Nigeria. This is the first WTP study in Akwa Ibom state. Awareness of health insurance was very poor among the rural dwellers. Majority of the rural dwellers have not heard of health insurance and are not covered by any form of health insurance. However, majority of the respondents are willing to pay for a contributory social health insurance scheme.

The estimated WTP is less than the benchmarked amount for social health insurance for the country by the NHIS. This makes a case for government establishment of schemes for rural dwellers and subsidization of the schemes to ensure viability and sustainability of the pools. Government should consider raising funds using other innovative financing mechanisms to subsidize and ensure viability of the schemes. Future research on WTP in Akwa Ibom state should consider addressing the WTP of the informal sector.

The regression model predicting WTP by rural residents is as follows:

$$\begin{array}{l} y=2419+0.1763\;*\;\left(x1\right)+741.5\;*\;\left(x2\right) \end{array}$$

Where y is WT, x1 = breadwinner income, x2 = size of household.

## Data Availability Statement

The raw data supporting the conclusions of this article will be made available by the authors, without undue reservation.

## Ethics Statement

The studies involving human participants were reviewed and approved by Institutional Health Research Ethics Board of the University of Uyo Teaching Hospital, Akwa Ibom State. The patients/participants provided their written informed consent to participate in this study.

## Author Contributions

CA conceptualized the paper. IU, AB, EF, SA, UO and OO contributed to the design of the study. EU organized the database. EN and CA performed the statistical analysis. CA wrote the first draft of the manuscript. EN wrote sections of the manuscript. SA made substantial contributions to the questionnaire design and data acquisition. All authors contributed to manuscript revision, read, and approved the submitted version.

## Conflict of Interest

The authors declare that the research was conducted in the absence of any commercial or financial relationships that could be construed as a potential conflict of interest.
